# Interesting Case in Prof. Hebra’s Lecture Room

**Published:** 1872-01

**Authors:** 


[From Boston Medical and Surgical Journal.]

### INTERESTING CASE IN PROF. HEBRA'S LECTURE ROOM.

We are indebted to Dr. P. O'Connell, of this city, for an account of a wonderful case of tattooing seen in Prof. Hebra's wards. We give it in his own words:—

"VIENNA, October 25th, 1871.

"As I entered the lecture room of Prof. Hebra to-day, a short time before his lecture was over, to await the arrival of Dr. Neumann, who followed him in the use of the same room, I had an opportunity to see an exhibition such as I never saw before, and I believe such as very few persons in the civilized world have seen as yet. Standing upon the demonstration pedestal was a man of about 5 feet 9 inches, perfectly naked, and of a remarkably fine physique, on exhibition before the class. His form, attitude and general appearance were such that at the first glance I supposed the figure to be a bronze statue; one of the masterpieces of some first-class artist who had used imagination to assist nature, and had produced a fine model of manly development. Vigorous, muscular, in the prime of life, his form alone would have made him an object of interest; while in addition to this there was a coloring to his skin which no doubt assisted the herculean symmetry of his form in giving me my first impression in reference to his being a bronze figure. He was tattooed completely from head to foot, from top to toe. There was not a square inch that was free from the coloring, and the work had been done in the most beautiful style imaginable. The skin presented a very handsome appearance, far more beautiful, I believe, than any leopard's skin can be, and having an effect like the elegant tracery of an exceedingly rich cashmere shawl; only that the coloring was done with indigo principally, with enough red inserted here and there to give it effect. His whole body, as it presented itself to view, was "a work of art;" and when one pauses to consider the immense amount of labor and the severe torture he must have undergone while this was performing, it will be easy to credit the statement that it was not a voluntary submission on his part, that made him the subject of the artist's skill. So far as I have been able to learn his story, it is this: That his name is George Constantine. He is a Greek by birth, and, in company with maraudors, organized into a band of robbers, entered Chinese Tartary, for the purpose of committing depredations and robbing a gold mine. He, I presume, was one of the leaders of the gang. The exhibition proved an unfortunate undertaking for some of them, however, as they were taken prisoners; and this man, in company with a few others—among them an American and a Spaniard—was ordered by the ruler of the country to be branded in this manner, so that everybody, whithersoever he went, would know him to be the greatest rascal in the world. The coloring on the palms of the hands consists of letters beautifully made, stating his case. That he did not recognize the difference between 'meum and tuum;' that he was the greatest rascal and thief in the world, &c. &c. &c., and warning people everywhere to beware of him. (This is the interpretation. The letters, of course, are not of our style or language.)

"It took three months of constant work to finish the job on him, working continually every day from morning till night; and his vigor of constitution must have been remakable to have enabled him to survive it. His comrades succumbed, whether during the operation, or subsequently, I do not know. The indigo was pricked into the skin, as one may readily imagine, without any regard for his feelings; and he says, in his profane vulgarity, that his sufferings exceeded those of 'Jesus Christ.'

"As I have said already, I do not think that it would be possible to find a square even one-quarter of an inch in size upon the surface of his body free from coloring. And on his head, conjecture said there must have been something more fearful still, as he kept the whole of his scalp covered with a black, Malay-looking swipe, which no persuasion could prevail upon him to remove.

"The different designs were not, each, more than a few inches in size, and they were representations of elephants, lions, tigers, birds of all kinds, with letters worked in between, referring, I presume, to his wickedness, each design being in itself a work of art, and the effect of the whole (with its thin outlines of the natural skin, tinted here and there with red, and winding its tortuous course all over the body) being very beautiful. No part of his body, however private, was spared; and when he extended his arm over his head, the symmetry of design and the same elegance of execution showed in the axilla, as in the more noticeable and exposed parts of his body. A couple of dragons ornamented, and glared at each other across his forehead. His cheeks had received their allowance of pigment, and his rough, wiry beard half-concealed and half-disclosed the labor that had been expended on the ornamentation of that part of his person. Altogether, he was a wonder to look upon; and with his dark, sinister eye glaring out from under his black head-covering, his muscular, finely-developed form, and the history connected with his case, one would recognize in him at once an incarnation of the legendary brigand chief or pirate captain; and I believe that the warning he carries upon his skin deserves to be heeded, 'Beware of him!' He is a wicked rascal, and is capable of being a fiendish one.

"The fellow dresses in a piratical way, and does not receive any addition to his looks from his clothing. He has lately escaped from the scene of his punishment, and I think has an idea that his strange and fearful ordeal may be converted into a means of making his fortune now, exhibiting himself. It is five years since the deed was done. His body swelled up very much at the time, was very sensitive to the weather, and continues somewhat sensitive to the weather even now. While the artist was at work, it was necessary to chain him down. I have no doubt but that he intends to exhibit himself as a means of getting a living. He intimated as much, referring to England and America as places that would pay him well—and thus in time our countrymen may have an opportunity to see him."

